# Implicit signals in small group settings and their impact on the expression of cognitive capacity and associated brain responses

**DOI:** 10.1098/rstb.2011.0267

**Published:** 2012-03-05

**Authors:** Kenneth T. Kishida, Dongni Yang, Karen Hunter Quartz, Steven R. Quartz, P. Read Montague

**Affiliations:** 1 Human Neuroimaging Laboratory, Computational Psychiatry Unit, Virginia Tech Carilion Research Institute, 2 Riverside Circle, Roanoke, VA 24018, USA; 2 Wellcome Trust Centre for Neuroimaging, 12 Queen Square, WC1N 3BG London, UK; 3 Department of Neuroscience, Baylor College of Medicine, One Baylor Plaza, Houston, TX 77030, USA; 4 Graduate School of Education and Information Studies, University of California, Los Angeles, Los Angeles, CA 90095, USA; 5 Social Cognitive Neuroscience Laboratory, Humanities and Social Sciences and Computation and Neural Systems Program, California Institute of Technology, 228-77, Pasadena, CA 91125, USA

**Keywords:** intelligence quotient, cognitive capacity, implicit signaling, small group interaction, prediction error, functional magnetic resonance imaging

## Abstract

Measures of intelligence, when broadcast, serve as salient signals of social status, which may be used to unjustly reinforce low-status stereotypes about out-groups' cultural norms. Herein, we investigate neurobehavioural signals manifest in small (*n* = 5) groups using functional magnetic resonance imaging and a ‘ranked group IQ task’ where implicit signals of social status are broadcast and differentiate individuals based on their expression of cognitive capacity. We report an initial overall decrease in the expression of cognitive capacity in the small group setting. However, the environment of the ‘ranked group IQ task’ eventually stratifies the population into two groups (‘high performers’, HP and ‘low performers’, LP) identifiable based on changes in estimated intelligence quotient and brain responses in the amygdala and dorsolateral prefrontal cortex. In addition, we demonstrate signals in the nucleus accumbens consistent with prediction errors in expected changes in status regardless of group membership. Our results suggest that individuals express diminished cognitive capacity in small groups, an effect that is exacerbated by perceived lower status within the group and correlated with specific neurobehavioural responses. The impact these reactions have on intergroup divisions and conflict resolution requires further investigation, but suggests that low-status groups may develop diminished capacity to mitigate conflict using non-violent means.

## 1. Introduction

People place great value on measures of their intelligence and work diligently to increase these scores in efforts to increase their social and professional status. In humans, one of the most conspicuous measures of intelligence is the intelligence quotient (IQ). To be ‘top of the class’ means that one is by definition *not* at the bottom. This differentiation of class membership can establish a stable hierarchy of power and may set a stage for inter-group conflict of varying magnitude. Indeed, the capacity to sense and act upon one's relative ranking within a group has played a major role in the evolution of social creatures [1–4]. The effects of group membership and ingroup/outgroup distinctions have dramatic effects on the expression of individuals' actions and beliefs [5], and may have important consequences on processes involved in moral decision-making. Indeed, the role of the individual, the group and the association between cognitive capacity and the ability to choose the ‘morally correct’ course of action has been argued [6].

Investigations of the neural basis of intelligence have shown the importance of activity in the lateral prefrontal cortex (LPFC) and other brain areas [7–9] in explaining individual IQ differences, while some studies correlate regional volumetric differences with heritable differences in IQ [10,11]. These studies suggest biologically based stability in an individual's expression of IQ. In contrast, behavioural studies show that simply framing the test-taker's environment with explicit or implicit cues about the test-taker's stereotyped social status can modulate one's expression of IQ [12–15]. The neurobiological basis of this effect is unknown; however, the signals engendered by the stereotype threat may be a subset of a broader class of signals that affect the expression of IQ.

**Figure 1.**
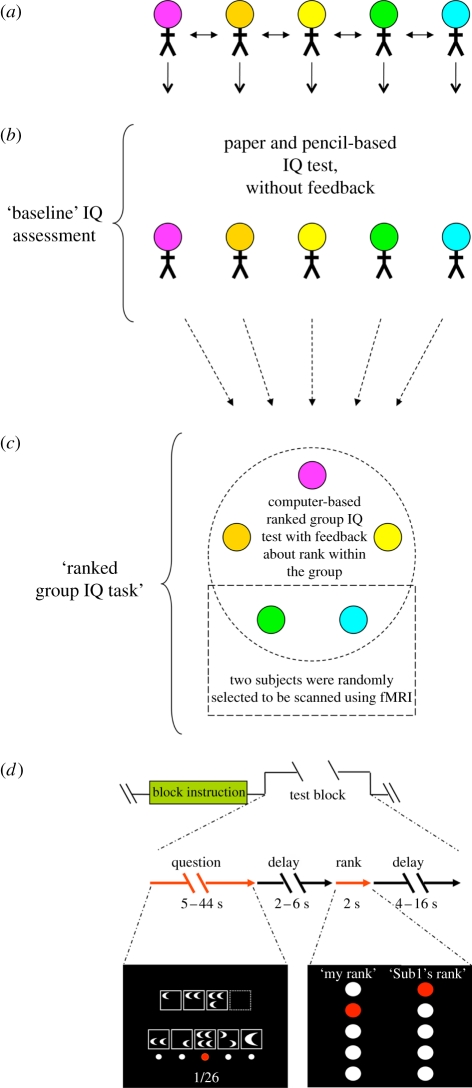
Five-person ranked group IQ paradigm. (*a*) Five subjects are recruited per group experiment. Subjects are introduced to each other by name prior to the beginning of the task. (*b*) A baseline IQ (*P*_IQ_) is assessed prior to the group scan: subjects answer questions from Cattell's culture fair intelligence test (questions from form 3A) using a pencil and paper—no feedback is given to the subjects during that time. The test is taken in an open room and all members of the group are present. Subjects' individual scores are not calculated until the end of the ranked group IQ task (see below). (*c*) Following the completion of the paper and pencil IQ task, the five-person group then participates in a five-person ranked group IQ task. During this task, subjects answer the same questions at the same time and are given feedback in the form of a ranking within the group of five, as well as the rank of a pseudorandomly selected member of the group. This test contains a total of 92 trials (one question per trial). The questions are modified from Cattell's culture fair intelligence test, forms 2A and 2B, and contain four types of questions that appeared in four corresponding blocks. Two of the five subjects are randomly selected to have their brains scanned using fMRI during this portion of the experiment. (*d*) Four blocks of thirteen questions are presented in a random sequence. Each block begins with an instruction screen (‘Block instruction’). The questions within a block are then presented in random sequence (‘Test block’; an illustrative example of a question screen is shown in the bottom left). Feedback about each subjects' performance is presented following each completed test question. Feedback is given in the form of a rank within the group, which is calculated based on the number of questions answered correctly out of the previous ten. The rank reveal screen (bottom right) consists of each subject's own rank (‘My rank’) and a pseudorandomly selected subject's rank (‘*Sub1*'s rank’ where *Sub1* is actually a pseudorandomly selected subject's first name). All participating subjects see their own rank and the same randomly selected subject's name and rank on each rank reveal screen.

Previous behavioural studies suggest that society-level signals about social status (i.e. stereotypes) are harmful to individuals' intellectual performance [14,15], yet it is unknown whether objective signals about rank within a small group can produce a similar harmful effect. We investigated the effect of dynamically evolving and objectively assessed signals about one's cognitive capacity on the expression of IQ within small groups (five people) and measured associated brain responses using blood oxygenation level-dependent (BOLD) imaging. Our paradigm, the ‘ranked group IQ task’ (figure 1), gave feedback to participants (*n* = 70) about their relative rank based on the group's recent test performance (the trailing 10 questions) and updated as the computer-based test progressed (92 questions total, modified from Cattell's culture fair intelligence tests [16], see §2 for details). This paradigm provides subjects the opportunity of upward and downward mobility throughout the task. Following every trial, the computer display showed each subject's personal rank privately and one randomly chosen subject's rank (figure 1*d*). Prior to the ranked group IQ task we determined a ‘baseline IQ’ (*P*_IQ_) for all subjects using a pencil and paper version of the test, which was taken without feedback (figure 1*b*). Demographic information for the participants is summarized in table 1.

**Table 1.** Demographics.

gender^a^	total (*n* = 70)^b^: male 49%, female 51%
	scanned (*n* = 27): male 52%, female 48%
	scanned HP (*n* = 13): male 10/13^a^, female 3/13
	scanned LP (*n* = 14): male 3/14, female 11/14^a^
age (mean ± s.e.)	total: 25.5 ± 0.6 (minimum 18, maximum 49)
	scanned: 25.1 ± 0.7 (minimum 21, maximum 35)
	scanned HP: 24.7 ± 0.7 (minimum 22, maximum 31)
	scanned LP: 25 ± 1 (minimum 21, maximum 35)
ethnicity	total: Caucasian 55%, Asian 25%, Hispanic 12%, African American 8%
	scanned: Caucasian 63%, Asian 11%, Hispanic 11%, African American 15%
	scanned HP (13): Caucasian 46%, Asian 15%, Hispanic 23%, African American 15%
	scanned LP (14): Caucasian 71%, Asian 0%, Hispanic 7%, African American 21%

^a^The distribution of males and females among the high- and low-performing scanned groups is significant, two-tailed *p*-value = 0.007, Fisher's exact test.

^b^Three subjects were excluded from analysis. Two of them had computer technical problems. The third one, who was also scanned, failed to complete the paper and pencil test.

## 2. Experimental procedures

### (a) Subjects

Seventy subjects were recruited from the Texas Medical Centre (Houston, TX, USA) or California Institute of Technology (Pasadena, CA, USA). All subjects were informed that they would be answering questions directly taken or modified from standard IQ tests. Subjects gave informed consent in accordance with the Institutional Review Board at Baylor College of Medicine, Houston, TX, USA (BCM) or California Institute of Technology (Caltech). Two subjects who experienced computer problems during the experiment were excluded from the analysis. BOLD imaging data were collected from 28 out of the remaining 68 subjects at BCM. We excluded one scanned subject from further analysis because they failed to complete the paper and pencil IQ assessment (baseline IQ score). In total, we analysed behavioural data from 67 subjects, of which 27 were scanned.

### (b) Baseline intelligence quotient assessment (P_*IQ*_)

Prior to the group portion of the task, all subjects were tested for their baseline IQ using a paper and pencil-based IQ test (Cattell's culture fair intelligence test, form 3A). No feedback was given to the subjects, and their performance on this test was not determined until after the completion of the ranked group IQ task.

### (c) Ranked group intelligence quotient task

Questions in the ranked group IQ task were taken from the Cattell's culture fair intelligence test, forms 2A and 2B. Permission to use and modify the tests was obtained from Institute for Personality and Ability Testing, Inc. (IPAT). To determine the difficulty of each individual question and develop the computer-based IQ task, a separate cohort of 93 subjects was recruited in a pilot study. Question difficulty was defined as the percentage of subjects getting a question wrong (for the easiest question difficulty will be 0, and for the hardest question the difficulty will be 1, i.e. 100% of the participating subjects got it wrong). In the pilot study, subjects took a paper and pencil test first and a timed computer-based IQ task. In the computer task, each type of questions was grouped and presented in blocks. Questions within each block came out in random sequences. Tests were also timed in blocks. Time limits for each block were calculated based on the time for corresponding paper and pencil tests plus 2 or 5 additional seconds for each question (no difference in performance was found between giving 2 or 5 s, so the two conditions were combined). The average time spent and the standard deviation of the mean were calculated for each question and were used in generating the five-person computer task parameters in the present study.

For the ranked group IQ task, we used 92 questions (13 questions from four different categories). Questions were scanned from the original tests and displayed on a monitor. All questions are multiple-choice. The four types of questions appeared as four corresponding blocks; the order of blocks was randomized, and each block began with an instruction screen signalling the new category of questions. Subjects were allowed up to 20 s maximum for the block instruction screen and were instructed to push a key when they were ready to proceed. Questions within each block appeared in a random sequence. Each question was revealed to all five subjects at the same time. Subjects used one hand to highlight their answer (selected from a set of multiple-choice options) and the other hand to submit their final selection. For scanned subjects, the hand used for submitting or choosing was counterbalanced. If subjects did not press the submit button before a predetermined time limit, then the item that was highlighted at that time was automatically submitted; the time limit for each question was determined from the above-mentioned pilot study (mean + 2 × s.e.). After all answers were submitted and a 2–6 s randomized delay, subjects were shown their own relative rank within the group of five and the rank of a pseudorandomly chosen member of the group (each subject was chosen 18 times, 5 × 18 = 90, the first two rank reveal screens included only each subject's own rank). The two ranks appeared randomly on the two sides of the screen and remained on the screen for 2 s. Subjects then saw a blank screen for 4–16 s before the next question came up. The entire 92-question ranked group IQ task was run in one continuous session. Between 1416 and 1617 functional scans were obtained for each scanned subject, mean = 1517 and s.d. = 11. The functional scans of one subject were discarded as explained above, due to the lack of a baseline IQ assessment. All other functional scans were used in the analysis. Prior to taking the ranked group IQ task, subjects were told, ‘…you will receive feedback on how you and other people are doing; the feedback will be given in the form of rankings’. The rules of rank calculation were explained to subjects (see §2*d*). After the computer IQ task, subjects filled out two questionnaires, the abbreviated NEO five factor personality inventory (60-question version) and the inventory of interpersonal problems. Each subject received $40 before leaving the laboratory.

### (d) Rank determination

Rank was computed according to the following algorithm: (i) determine the fraction of correct answers of the last ten questions, (ii) for the first nine questions, total correct answers were used in place of the ratio, (iii) the highest ranks are given to the subjects with the highest score, and (iv) if there is a tie, then subjects share the highest possible rank given their performance in the group.

### (e) Performance-based categorization of subjects

**Figure 2.**
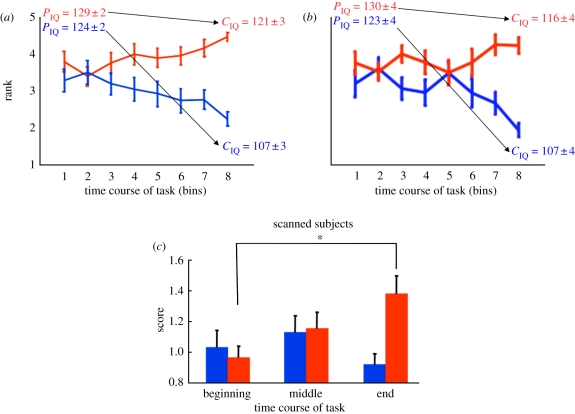
Group intelligence quotient (IQ) task with ‘social status’ feedback demonstrates harmful influence on expressed IQ. (*a*) Subjects' final ranking identifies two groups with differentiated trajectories during the group IQ task. *x*-Axis: time course of the task, the experiment excluding the first 12 questions is divided into eight bins, each bin consisting of 10 trials; *y*-axis: rank as assessed in each of the eight bins. Two groups were defined by their rank at the end of the task (last bin): group 1 (*n* = 19) had a rank greater than the median (red), whereas group 2 (*n* = 20) had a rank less than or equal to the median (blue). Subjects in group 1 and group 2 were selected such that their baseline IQ scores (*P*_IQ_) were similar (i.e. group 1 and group 2 did not differ significantly on IQ scores derived from their pencil and paper-based test, ‘*P*_IQ,group#_ = mean ± s.e.m.’: *P*_IQ,group 1_ = 129 ± 2 versus *P*_IQ,Group 2_ = 124 ± 2, *p* = 0.06, two-sample *t*-test). According to the performance on the ranked group IQ task (*C*_IQ_), group 1 subjects' mean IQ was determined to be ‘*C*_IQ,group#_ = mean ± s.e.m.’: *C*_IQ,group 1_ = 121 ± 3, whereas group 2 subjects' mean IQ was determined to be *C*_IQ,group 2_ = 107 ± 3, which indicates a significant difference (*p* < 0.05). (*b*) A subset of scanned subjects (*n* = 27) showed similar rank changes in the group IQ task. Scanned subjects were divided into two groups by their rank at the end of the task as mentioned above. High performers (HP; *n* = 13) had a rank greater than the median (red). Low performers (LP, *n* = 14) had a rank less than equal to the median (blue). The two groups had similar baseline IQ (*P*_IQ_ = mean ± s.e.m.): *P*_IQ_ = 130 ± 4 in HP group versus *P*_IQ_ = 123 ± 4 in LP group, *p* = 0.2, two-sample *t*-test; however, they showed a significant difference (*p* < 0.05) in expressed IQ by the end of the ranked group IQ task—‘*C*_IQ (HP or LP)_ = mean ± s.e.m.’: *C*_IQ,HP_ = 116 ± 4, *C*_IQ,LP_ = 107 ± 4. (*c*) High performing subjects improve their performance during the ranked group IQ task. *x*-Axis: the experiment is divided into three epochs (beginning, middle and end); *y*-axis: normalized, difficulty adjusted cumulative scores for ranked group IQ task performance. Only fMRI-scanned subjects (*n* = 27) are plotted here and in subsequent analyses. As labelled in figure 2*b*, subjects ending with ranks > median define one group (red bars, *n* = 13, ‘HP’). Those ending with ranks ≤ median define the second group (blue bars, *n* = 14, ‘LP’). All subjects analysed here possessed similar baseline IQ scores. Initially, both groups perform poorly (see ‘beginning’ scores for both low and high performing groups). By the end of the ranked group IQ task, the high performing subjects (i.e. highest final ranks; red bars) steadily increased their performance (compare red bars at the beginning, middle and end) compared with the LP (i.e. lower final ranks; blue bars). Low performing subjects do not change their performance during the experiment (compare blue bars at the beginning, middle and end). Bar height and error bars indicate mean ‘difficulty adjusted score’ + s.e.m. Repeated-measure ANOVA showed significant group × time effect (*p* < 0.01) but no effect of group or time. *p < 0.05, post hoc with Bonferroni correction for multiple comparisons.

Following the completion of the ranked group IQ task, we performed a median-based categorization of subjects into two analysis groups; we placed individuals with a final average rank of the last 10 trials greater than the median into one group ‘group 1’ and those with a final average rank less than or equal to the median into a second group ‘group 2’. We excluded an equal number of individuals with the highest and lowest *P*_IQ_ before the separation such that the resulting two groups would be comparable (similar sample size and baseline IQ). For the initial behavioural analysis, our median-based categorization resulted in the analysis of 19 subjects for group 1 and 20 subjects for group 2. By design, these two groups did not differ in baseline IQ scores, but were categorically different based on their final rank in the ranked group IQ task (figure 2*a*). We could not scan all participants, so we randomly assigned two from each group of five to be scanned, whereas the other three participated at isolated computer terminals. We performed a similar performance-based categorization of the scanned subjects (*n*_scanned_ = 27) into two groups (*n* = 13 HP and *n* = 14 LP).

### (f) Behavioural data analysis

We used SPSS (SPSS Inc., Chicago, IL, USA) or Matlab7 (The Mathworks, Natick, MA, USA) for behavioural data analysis. Two IQ scores were estimated for each subject: *P*_IQ_ from the paper and pencil test and *C*_IQ_ from the five-person ranked group IQ task. Raw scores were converted to IQ using conversion tables provided by IPAT. Rank revelations following questions 13–92 were grouped into eight bins with 10 questions in each bin. Mean rank in each bin was calculated for subjects in each group. Rank revelations 1–12 were excluded because those initial ranks were based on subjects' performance on a small number of questions that appeared at the beginning of the experiment, and were thus likely to be more variable and less reliable. To compare the scores of two groups of subjects at the first, second and third epochs of the experiment (figure 2*b*), a repeated-measure two-way ANOVA was used. Difficulty adjusted scores for each subject in the three parts of the experiment were calculated: Score_*ij*_ = ∑ (result_*ij*_ × difficulty_*ij*_)/size(result_*ij*_); *i* = 1, 2, … 27; *j* = 1, 2, 3. Question difficulty was defined in a separate pilot study as described above. The scores were then normalized to the mean score of all subjects in the first epoch of the experiment. Post hoc tests were performed using Bonferroni adjustment.

### (g) Functional magnetic resonance imaging data acquisition and analysis

T1-weighted scans were acquired on Siemens 3T Allegra scanners at BCM. A high-resolution (0.5 × 0.5 × 1 mm) anatomical image was first acquired. Functional images were acquired with a repetition time of 2 s, echo time 25 ms, flip angle 90° in 37 interleaved slices (3.4 × 3.4 × 4 mm). Slices were angled approximately 30° from the anterior commissure–posterior commissure line. Using statistical parametric mapping (SPM2, Institute of Neurology, London, UK), functional images were adjusted to correct slice timing, realigned, co-registered to T1 anatomical image, normalized to Montreal Neurological Institute (MNI) coordinates and smoothed using an 8 mm Gaussian kernel. Data were high-pass filtered at 128 s.

General linear model (GLM) and random-effect analysis were performed using SPM2. The following conditions were included in the GLM: (i) displaying of instruction screens, (ii) displaying of rank, with a first-order modulatory parameter *ΔR* (*ΔR*_0_ = 0, *ΔR*_*n*_ = *R*_*n*_ − *R*_*n*__−__1_ for *n* = 2:92, *R* is subject's own rank), (iii) displaying of question, (iv) key presses for answer selection, (v) key press for answer submission, and (vi) parameters for head movements (*x*, *y*, *z*, pitch, yaw, roll). For each condition, boxcar functions for each event were convolved with a fixed hemodynamic response function. The durations of the boxcars for instructions, rank displays, question displays are indicated in figure 1*d*. Key presses for answer selection and answer submission are modelled as an impulse function, durations are 0 s. On average, scanned subjects pressed the submit button 52 times (for 92 questions) with s.d. = 4. In the event that the submit button was not pressed, the highlighted answer was selected for the subject (this was instructed and understood by all participating subjects). The average time delay between answer selection and submission is 2.8 s (range: 0.3–21.3 s).

Random-effects analysis was performed on parameter estimates from all subjects. xjView was used to visualize contrast images (http://www.alivelearn.net/xjview8/). At *p* < 0.0001 (uncorrected) with cluster size ≥ 10, bilateral nucleus accumbens and bilateral anterior cingulate cortex responses correlate positively and negatively with *ΔR*. Clusters of voxels identified by GLM regression were defined as regions of interest (ROI) and further analysed for comparisons across groups and across temporal epochs of the task.

A second GLM was constructed to identify regions showing differential activity in the course of the experiment. The experiment was divided into three epochs: the first included questions 1–30, the second 31–60 and the third 61–92. Rank revelations from the three sections were modelled as three conditions without a modulatory parameter. Question displays were also modelled as three corresponding conditions. Instructions, key presses and head movements were modelled the same way as in the first GLM. Because we expect that the effect of rank will develop over the course of the experiment, four specific contrasts were made: (i) for rank reveal, last epoch > first epoch; (ii) for rank reveal, last epoch < first epoch; (iii) for question reveal, last epoch > first epoch; and (iv) for question reveal, last epoch < first epoch. Bilateral amygdala was identified from ‘rank reveal, last epoch < first epoch’ contrast with *p* < 0.00001, uncorrected, and cluster size ≥ 10. Dorsolateral prefrontal cortex (DLPFC) was identified from ‘question reveal, last epoch > first epoch’ contrast with *p* < 0.0001, uncorrected, and cluster size ≥ 10. Identified voxels in amygdala and DLPFC were included in the ROI analysis. (See tables 2 and 3 for complete list of regions identified in the contrasts.) Preprocessed signals from ROI were averaged, detrended to remove task unrelated signal drift and converted to percentage signal change. Signal at time points of interest was obtained by aligning all instances of the specific condition. Linear interpolation was used when the time points fell between two scans.

**Table 2.** Regions differentially activated at the beginning and the end of the experiment. ACC, anterior cingulate cortex; PCC, posterior cingulate cortex. Contrast: ‘rank beginning’ < ‘rank end’ did not reveal any cluster with number of voxels ≥ 10 and *p* < 0.00001.

structure	left/right	Talairach coordinates			*Z*-score	cluster size
		*x*	*y*	*z*		
contrast: rank_beginning > rank_end (*p* < 0.00001, uncorrected, cluster size ≥ 10)						
amygdala	L	−20	−8	−16	5.31	17
amygdala	R	24	−4	−20	5.43	22
midbrain	R	8	−8	−8	5.04	19
inferior frontal gyrus (BA9)	L	−44	4	28	5.37	35
contrasts: question_beginning > question_end (*p* < 0.0001, uncorrected, cluster size ≥ 10)						
superior/middle frontal gyrus (BA9 BA10)	L	−8	16	32	4.47	30
ACC	L	−28	44	28	5.14	22
contrasts: question_beginning < question_end (*p* < 0.0001, uncorrected cluster size ≥ 10)						
PCC, precuneus (BA23 BA30 BA7)	R	4	−56	20	4.96	57
inferior/middle frontal gyrus (BA46)	R	48	28	20	4.31	17

**Table 3.** Regions responding to positive or negative group status change. ACC, anterior cingulate cortex.

structure	left/right	Talairach coordinates			*Z*-score	cluster size
		*x*	*y*	*z*		
contrast: negative correlation with *ΔR* (*p* < 0.0001, uncorrected, cluster size ≥ 10)						
ACC	L	−8	20	32	4.50	16
ACC	R	12	20	32	4.64	25
contrast: positive correlation with *ΔR* (*p* < 0.0001, uncorrected, cluster size ≥ 10)						
nucleus accumbens	L	−12	4	−12	5.18	34
nucleus accumbens	R	12	8	−12	5.08	33

## 3. Results

### (a) Performance-based stratification of intelligence quotient-matched subjects

We hypothesized that subjects' performance would be differentially affected by group rank feedback. Because subjects had no knowledge of their relative IQ at the start of the experiment, any rank effect should gradually develop over the course of the ranked group IQ task, with the maximum difference between HP and LP at the end of the experiment. To determine the difference between HP and LP in the ranked group IQ task, we selected individuals whose baseline IQ was similar (mean *P*_IQ_ = 126) and performed a median filter at the end of the ranked group IQ task (figure 2*a* and see §2 for detail). Those individuals who ended with a rank greater than the median were placed into our analysis category labelled group 1 (figure 2*a*, red trace, *n* = 19), while those with a rank less than or equal to the median were placed into group 2 (figure 2*a*, blue trace, *n* = 20). Individuals with the highest and lowest baseline IQ were excluded before applying the median filter so that the resulting two groups did not significantly differ in their pencil and paper-based IQ scores. A minimum of 14 subjects with highest IQ (mean = 143) and 14 subjects with lowest IQ (mean = 101) were excluded such that the resulting two groups possessed statistically similar baseline IQs (mean *P*_IQ_: group 1 *P*_IQ_ = 129 ± 2, range: 113–137; group 2 *P*_IQ_ = 124 ± 2, range: 117–137, *p* = 0.06, two-sample *t*-test).

### (b) Ranked group intelligence quotient task initially harms the performance of all subjects

Initial inspection of the effect of taking the test in the ranked group IQ paradigm suggests that the performance of group 2 was significantly harmed. By the end of the ranked group IQ task, group 2's score dropped an average 17.4 points (mean ranked group IQ score (*C*_IQ_) = 107 ± 2), which is a significant drop in performance compared with their baseline performance (*p* < 0.0001, one-sample *t*-test), whereas the performance of group 1 members remained relatively intact (mean *C*_IQ_ = 121 ± 2, a drop of 8 ± 4 points, *p* = 0.04, one-sample *t*-test, which is significantly less than the drop expressed by group 2, *p* = 0.04, one-tailed two-sample *t*-test). Figure 2*a* shows the history of the average ranks for group 1 (red trace) and group 2 (blue trace) members throughout the ranked group IQ task. The final point in the trace defined the separation of the two groups; however, looking back into their respective histories shows that in early stages these individuals were indistinguishable by early rankings (figure 2*a*), which is consistent with their indistinguishable *P*_IQ_.

### (c) Performance-based stratification of functional magnetic resonance imaging-scanned intelligence quotient-matched subjects

A subset of the entire subject pool (28 subjects of 70 total, or two out of every group of five) was randomly selected to be scanned using fMRI. Our remaining analyses focus on the connection between the observed behavioural changes (test performance) and measured brain responses. One scanned subject failed to complete the paper and pencil test and was thus excluded from further analysis. The remaining 27 subjects are divided into group 1 (HP: 13 scanned subjects with final rank > median) and group 2 (LP: 14 scanned individuals with final rank < median) categories as described for the larger group analysis. These two groups do not differ in their baseline IQ scores (*P*_IQ_ = 130 ± 4 versus *P*_IQ_ = 123 ± 4, *p* = 0.2, two-sample *t*-test). Among these 27 subjects, the same pattern of rank change as the larger pool of subjects was observed throughout the task (figure 2*b*).

Assessment of the performance of high- versus low-performing groups during early middle and late stages of the task using difficulty adjusted scores (figure 2*c* and table 4) shows that initially the performance of both groups was diminished (figure 2*c*, early). As the task progressed (figure 2*c*, middle and late), the ‘HP’ gradually improved (figure 2*c*, rising progression of red bars) whereas the ‘LP’ remained low (figure 2*c*, unchanging progression of blue bars). Repeated-measure ANOVA with group as a between-subject factor and time as a within-subject factor showed significant group × time effect (*p* = 0.007) but no group (*p* = 0.17) or within-subject time effect (*p* = 0.14). Taken together, these results demonstrate that placing individuals into a socially ranked testing paradigm can initially harm the performance of everyone. Additionally, the differentiation of the two groups here reflects a sustained suppression of the performance of the LP (figure 2*b*) and not an overall heightened performance of HP; rather, the HP start with suppressed performance and approach their baseline (*P*_IQ_) performance levels.

**Table 4.** Difficulty adjusted scores and brain signals over the course of the experiment.

group	time	difficulty adjusted scores	amygdala signal^a^	DLPFC signal^b^
high performers	beginning	0.97 ± 0.07	0.15 ± 0.02	0.10 ± 0.04
	middle	1.2 ± 0.1	0.11 ± 0.03	0.15 ± 0.03
	end	1.4 ± 0.1^c^	0.01 ± 0.03^d^	0.26 ± 0.06^e,f^
low performers	beginning	1.0 ± 0.1	0.13 ± 0.04	0.10 ± 0.02
	middle	1.1 ± 0.1	0.10 ± 0.03	0.14 ± 0.04
	end	0.92 ± 0.07^g^	0.10 ± 0.03^h^	0.16 ± 0.03^i^

^a^Amygdala signal was the average of 4–10 s after rank reveal from regions identified in the contrast rank_beginning > rank_end.

^b^r-LPFC signal was the average of 10–18 s after question reveal from regions identified in the contrast ques_beginning < ques_end.

^c^Different from beginning in HP, one-way ANOVA in HP, *p* = 0.017 with Bonferroni correction.

^d^Different from beginning in HP, one-way ANOVA in HP, *p* = 0.037 with Bonferroni correction.

^e^Different from middle in HP, one-way ANOVA in HP, *p* = 0.011 with Bonferroni correction.

^f^Different from beginning in HP, one-way ANOVA in HP, *p* < 0.0001 with Bonferroni correction.

^g^Smaller than end in HP, one-tailed two-sample *t*-test, *p* = 0.0011.

^h^Larger than end in HP, one-tailed two-sample *t*-test, *p* = 0.011.

^i^Smaller than end in HP, one-tailed two-sample *t*-test, *p* = 0.048.

### (d) Gender discriminates scanned high- and low-performing groups

We hypothesized that individual differences in the experienced trajectory of rank change during the experiment or differences in personality characteristics may be associated with the differential responses to rank between the two groups. HP and LP were not different in the number of instances of observed rank improvement or decrement or in the number of instances they saw their own rank as better or worse than the publicly revealed rank in the first half of the experiment (electronic supplementary material, table S1). To determine the effect of personality characteristics, we assessed scores on the NEO five factor personality inventory and the inventory of interpersonal problems. Comparison of HP versus LP on these scales did not find difference between these two groups along any of the axes in the two questionnaires (electronic supplementary material, table S2). However, in the scanned subject pool (*n* = 27), gender was a significant factor in the separation between HP and LP groups (table 1). Ten of the 13 HP individuals are male, whereas 11 of the 14 LP individuals are female. We used Fisher's exact test to determine a two-tailed *p*-value of 0.007 for this particular distribution of gender across the two categories. *A priori*, we did not expect differentiation between the sexes; therefore, we balanced the number of scanned male and female subjects. Other demographic variables (age and ethnicity) are not significantly different across the HP and LP groups (table 1). Next, we investigated the neurobiological correlates of this behaviour.

### (e) Neural correlates for the harmful effect of the ranked group intelligence quotient task

**Figure 3.**
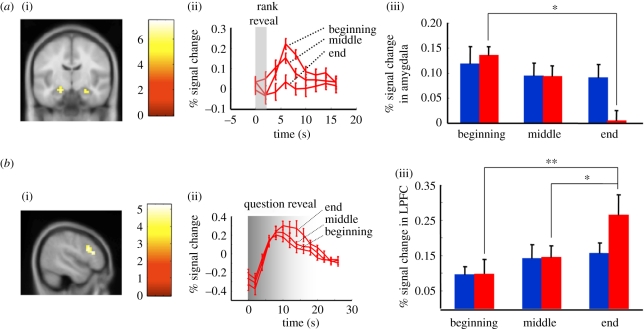
Associated brain responses in subjects scanned during the ranked group IQ task. fMRI-scanned subjects were divided into two groups as described in figure 2*b*: subjects (*n* = 27) were divided into two groups according to their final rank; subjects ending with ranks > median are labelled by red bars (*n* = 13, HP), those ending with ranks ≤ median are labelled by blue bars (*n* = 14, LP). Random-effects general linear model (GLM) analyses including all 27 subjects identified the amygdala and lateral prefrontal cortex as regions whose response changed during the time course of the ranked group IQ task. (*a*) BOLD responses in the amygdala decrease in high performing subjects. (i) A random-effects GLM analysis including all scanned subjects (*n* = 27) with the contrast: ‘rank_beginning’ > ‘rank_end’ identified bilateral amygdala. (ii) Time course of the amygdala response to the ‘rank reveal’ screen in HP (*x*-axis: time in seconds; *y*-axis: percentage change in the BOLD response; bars and error bars indicate mean + s.e.m.). At *t* = 0, the subjects' rank was displayed; the traces show the amygdala response at early, middle and end stages of the test in HP. Repeated-measure ANOVA showed significant within-subject time effect (*p* = 0.002) and time × group effect (*p* = 0.02), but no significant group effect (*p* = 0.49). (iii) HP (red bars) amygdala activity (peak 4–10 s after rank reveal) decreased at the end of the experiment compared with the beginning (*p < 0.05; post hoc with Bonferroni correction). In contrast, the amygdala response from LP (blue bars) showed no significant changes throughout the task. (*b*) BOLD responses in the LPFC increase in high performing subjects. (i) A random-effects GLM analysis with the contrast: ‘question_reveal_end’ > ‘question_reveal_beginning’ identified the right-lateral prefrontal cortex (r-LPFC). (ii) Time course of the r-LPFC response to the ‘question reveal’ screen in subjects from high performers (*x*-axis: time in seconds; *y*-axis: per cent change in the BOLD response; bars and error bars indicate mean + s.e.m.). At *t* = 0, a question was displayed; the traces show the r-LPFC response at early, middle and end stages of the test in the high performers. Repeated-measure ANOVA showed significant within-subject time effect (*p* < 0.0001) and time × group effect (*p* = 0.007), but no significant group effect (*p* = 0.45). (iii) High performers (red bars), r-LPFC activity increased (10–18 s after the question was revealed) at the end of the experiment compared with the beginning and middle (**p < 0.0001, *p < 0.05, post hoc with Bonferroni correction). In contrast, the r-LPFC response from LP (blue bars) showed no significant changes throughout the task.

**Figure 4.**
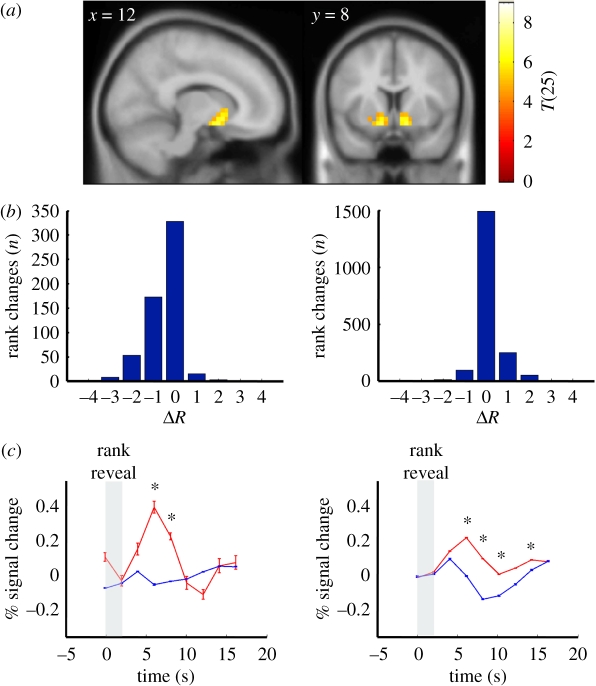
Changes in rank are associated with dynamic responses in the nucleus accumbens that are consistent with a prediction error signal. (*a*) Nucleus accumbens parametrically responds to positive changes in rank: a random-effects GLM analysis for responses that correlated with parametric changes in rank identified only the bilateral nucleus accumbens for positive changes in rank (random-effects, *n* = 27, regions with 10 or more voxels significant at *p* < 0.0001, uncorrected). (*b*) Positive and negative changes in rank are observed immediately following trials answered correctly or incorrectly: histograms showing the distribution of rank changes following incorrect (left) or correct (right) responses to test questions. Horizontal axis: change in rank (*ΔR*) following an incorrect answer (left) or a correct answer (right). Vertical axis: number of events (*n* = 92 trials × 27 scanned subjects = 2484 events, i.e. ‘rank changes’). Rank was calculated based on the performance of the last 10 trials, thus a subject's rank could change following every question and may not follow the performance of the last trial (i.e. an incorrectly answered question could precede an increase in rank (left) or a correctly answered question could be followed by a decrease in rank (right)). (*c*) BOLD responses in nucleus accumbens to changes in rank following incorrect (left) or correct (right) responses to test questions. Horizontal axis: time (seconds); vertical axis: BOLD response expressed as the percentage change from baseline following the revelation of one's own rank (vertical grey bar); red traces: BOLD responses (mean ± s.e.m.) in the nucleus accumbens associated with rank increases; blue traces: BOLD responses (mean ± s.e.m.) in the nucleus accumbens associated with rank decreases (blue traces). Although subjects did not have explicit feedback about whether they answered the last question correctly or incorrectly the responses observed in the nucleus accumbens are consistent with an expectation error over the effect of answering trials correctly and the effect it should immediately have on one's rank. Asterisk denotes significant difference at corresponding time points between red and blue traces (*p* < 0.05, **two-sample *t*-test).

Using BOLD imaging, we measured brain responses associated with the changes in performance during the ranked group IQ task. Twenty-eight subjects were scanned. One subject failed to complete the baseline IQ test and was excluded from further analyses. Data collected from the remaining 27 individuals were subjected to random-effects GLM analyses. GLM contrasts designed to compare the HP and LP groups' responses with either rank revelation or question revelation throughout the entire task did not identify significantly different activation with clusters with greater than 10 voxels at *p* < 0.005 threshold. However, we expected the effects of feedback about one's rank in the group to accumulate as the ranked group IQ task progresses. To test this hypothesis, we divided the task into early, middle and late epochs for GLM contrast analyses. Differences in the neural responses to the revelation of one's own rank (table 2 and figure 3*a*) or the revelation of test questions (table 2 and figure 3*b*) were determined between late and early epochs. Another GLM without dividing the experiment was used to investigate whole brain responses to changes in one's rank (table 3 and figure 4*a*).

### (f) Blood oxygenation level-dependent responses in the amygdala decrease in high performers as the task progresses

A GLM contrast comparing rank revelation at the end of the task compared with rank revelation at the beginning of the task revealed greater activity in bilateral amygdala during the early rounds of the task compared to late rounds (all scanned subjects included in the analysis, *n* = 27; table 2 and figure 3*a*(i)). Although a direct comparison of the two groups (HP versus LP) did not yield any significant clusters (likely owing to sample size, *n* = 13 and *n* = 14, respectively), further analysis comparing the time series of the BOLD response in the amygdala from HP individuals—during early, middle, and late rounds—showed a gradual decrease in the peak response (table 4 and figure 3*a*(ii)). LP individuals did not display this gradual change (table 4 and figure 3*a*(iii)). Repeated-measure ANOVA of all scanned subjects with time as a within-subject factor and group as a between-subject factor showed significant within-subject × time (*p* = 0.002) and time × group effects (*p* = 0.02), but no significant group effect (*p* = 0.49). During the earliest epoch of the task, amygdala responses following rank revelation did not differ between HP and LP groups (*p* = 0.6, two-sample *t*-test). However, in the latest epoch amygdala responses were significantly lower in HP (*p* = 0.01, one-tail two-sample *t*-test).

### (g) Blood oxygenation level-dependent responses in the lateral prefrontal cortex increase in high performers as the task progresses

We also performed a GLM contrast over all scanned subjects comparing question revelation at the end of the task compared with question revelation at the beginning of the task. This analysis revealed greater activity in right-lateral prefrontal cortex (r-LPFC) during the late rounds of the task compared with early rounds (table 4 and figure 3*b*(i)). Further analysis comparing the time series of the BOLD response in the r-LPFC of the HP group—during early, middle, and late rounds—showed a gradual increase in the response (table 4 and figure 3*b*(ii)). LP individuals failed to display this gradual change (table 4 and figure 3*b*(iii)). Repeated-measure ANOVA for all scanned subjects with time as the within-subject factor and group as the between-subject factor showed a significant within-subject × time effect (*p* < 0.0001) and time × group effect (*p* = 0.007), but no significant group effect (*p* = 0.45). Similar to the amygdala response, r-LPFC following question revelation at the earliest epoch is similar between HP and LP (*p* = 1.0, two-sample *t*-test). However, contrary to the amygdala response, r-LPFC responses are significantly greater in HP at the latest epoch (*p* = 0.048, one-tail two-sample *t*-test). The difference between the two groups in terms of performance, amygdala activation and r-LPFC activation is biggest towards the end of the task; it is possible that more subtle changes occurred earlier.

### (h) Blood oxygenation level-dependent responses in the nucleus accumbens are consistent with prediction error signals over expected changes in rank

Finally, we performed a GLM analysis using the amount of ‘change in rank’ as a parametric regressor on rank revelation for all subjects scanned. This analysis revealed greater activity in bilateral nucleus accumbens for positive changes in rank (figure 4*a* and electronic supplementary material, figure S1) and greater activity in the dorsal anterior cingulate cortex for negative changes in rank (electronic supplementary material, figure S1). The response in the anterior cingulate cortex is consistent with previous reports of this region responding to conflict and recruitment of alternative cognitive strategies [17,18].

The nucleus accumbens has previously been implicated as a site for dopamine mediated reward prediction error signalling during learning [19–23]. We hypothesized that BOLD responses in the nucleus accumbens could have been positive or negative prediction error signals over expectations about one's changes in rank during the ranked group IQ task. To test this hypothesis, we compared the BOLD response in all scanned subjects following one's change in rank when subjects answered the preceding question correctly versus incorrectly. Changes in rank were calculated based on the whole group's performance over the preceding ten questions; thus an individual's performance on the most recent question may be positively or negatively correlated with the most recently observed change in rank. Behavioural analysis shows that subjects typically observe negative changes in rank when they have just answered a question wrongly (figure 4*b*, left) and positive changes in rank when they have just answered a question correctly (figure 4*b*, right). However, there are many instances when a subject's rank changes in the opposite direction: positive change in rank following an incorrect answer and negative change in rank following a correct answer (figure 4*b*). Note that subjects did not have access to explicit knowledge about the correctness of their answers. Yet, analysis of the BOLD response in the nucleus accumbens is consistent with the postulate that subjects have expectations about their impending rank revelation. Figure 4*c* shows four features that suggest the response in the nucleus accumbens is consistent with an expectation error over subjects' belief about their own performance: (i) the nucleus accumbens responded strongest and positive when subjects answered a question incorrectly and their rank increased (i.e. ‘much better than expected’; figure 4*c*, left panel, red trace), (ii) the nucleus accumbens responded strongly and negative when subjects answered a question correctly and their rank decreased (‘worse than expected’; figure 4*c*, right panel, blue trace), (iii) the nucleus accumbens did not respond when subjects answered incorrectly and their rank decreased (‘as expected’; figure 4*c*, left panel, blue trace), and (iv) the nucleus accumbens responded positively to positive changes in rank following correctly answered trials; notice that this response is approximately half the magnitude observed in point 1 (‘better than expected’; figure 4*c*, right panel, red trace). This pattern of responses is consistent with expectation error signal over subjects' beliefs about their performance although they do not explicitly know the correctness of their answers. We tested this hypothesis in a secondary analysis where we included the magnitude in the change in rank and calculated the prediction error trial by trial for each subject and compared this value to the peak of the response in the nucleus accumbens. The correlation between the calculated prediction error and the observed nucleus accumbens response bolsters the interpretation above (Pearson's *r* = 0.1063; *p* < 10^−6^).

Previous work regarding reward-related brain activity has suggested that the ventral striatum is responsive to social comparisons [24]. In the ranked group IQ task, for each rank revelation, one pseudorandomly selected person's rank was displayed next to each subject's own rank. This public broadcasting was included to reinforce the notion of a socially broadcasted signal. In a GLM with public rank minus own rank as a first-order modulator of rank revelation, no brain regions showed statistically significant correlation with public-own rank (uncorrected threshold of *p* < 0.0001, voxel size > 10).

## 4. Discussion

Societal and cultural behaviours organize around a variety of signals generated and understood in the context of small groups. Such groups are susceptible to explicit signalling from inside and outside the group, but they are also sensitive to implicit signalling. One important signal in the context of a small group is social rank; however, the cognitive impact of changes in social rank is not well understood and almost nothing is known about the neural underpinnings. We have used a small group test-taking paradigm, the ranked group IQ task, to assess the effect of explicit signals about status within small groups on the expression of individual intelligence. We identified two separate groups using an end-stage median filter on overall performance within the cohort of tested subjects (figure 2*a*). Our approach is analogous to the social filters present in real life where the top performers advance based on presumed merit. Our results call into question the ability of these types of filters to select solely on the explicit filter criteria. Specifically, our analysis compared individuals with relatively high IQ scores (mean *P*_IQ_ = 126 compared with the general population mean IQ ∼ 100), and showed that a test environment where signals about social rank were broadcast initially suppressed the performance of everyone involved. This effect was stabilized in a subset of the members of the small group. In addition, we demonstrate responses in key brain regions that reveal covert differences in the expression of problem solving ability observed in the two groups.

Amygdala activation was high and remained high in the low-performing group (figure 3*a*) and has been thought to reflect fear and emotional arousal [25–28]. It has been shown that presentation of fearful faces or emotional scenes harms performance in working memory tasks [29,30]. The decrease of amygdala activation during rank revelation in HP subjects might reflect decreased fear or emotional arousal to being ranked, resulting in less detrimental effects on working memory required to perform well on this IQ task. Alternatively decreased amygdala activation in HP at the end of the task may result from observing better ranking relative to the LP subjects, who see lower ranks by the end of the task. We tested this hypothesis by assessing amygdala responses in instances when one's rank is better than the median compared to instances when one's rank is worse than the median (electronic supplementary material, figure S2*a*, *p* > 0.05, two-sample *t*-test). Another possibility is that the amygdala is responding to relative ranking between the individual and the other publicly revealed rank [31,32]. Amygdala responses in instances when one's rank is better than the publicly revealed rank is not different from amygdala responses when one's rank is worse than the publicly revealed rank (electronic supplementary material, figure S2*b*, *p* > 0.05, two-sample *t*-test). This lack of association between amygdala activation and relative ranks is consistent with the hypothesis that the observed decreasing amygdala response in HP is associated with decreasing fear, anxiety or emotional arousal associated with the social ranking during this task regardless of their current status (i.e. high or low rank). How the HP subjects achieve this is unclear and a subject for further investigation.

We also demonstrate that those individuals that overcome the initial decrement in performance show reduced amygdala activation during rank revelation and increased activity in the r-LPFC following test question presentation (figure 3*b*). Previous studies have shown that regions overlapping or close to the r-LPFC cluster are active when people work on similar kinds of IQ tasks or working memory tasks; these regions also show increased activation when task difficulty was increased [7,8]. Gray's study [8] using IQ tasks similar to ours showed activation of brain regions that partly overlap the above r-LPFC, with higher event-related activity in subjects with higher IQ and better scores. The difference between HP and LP r-LPFC responses is consistent with the hypothesis that the HP group is initially similar to the LP group (diminished in the small group setting), but recovers their HP performance level by the end of the task, whereas the LP group remains inhibited. Previous studies have linked prefrontal activity to emotion regulation [33]; thus, it is reasonable to hypothesize that increased r-LPFC activity could help to reduce fear or anxiety, which may be associated with increased responses in the amygdala. The direction of modulation between these two areas or whether there is an intermediate region influencing both areas is unclear at this time; possible hypotheses include: (i) top-down regulation of the amygdala by PFC activity could be involved in reducing a fearful or anxious state allowing greater concentration and performance on the IQ task, (ii) bottom-up modulation of r-LPFC activity by the amygdala directly could interfere with performance on the ranked group IQ task or (iii) an intermediate region could be involved in modulating both regions independently of one another.

Finally, we show responses in the nucleus accumbens to changes in social status that act like learning signals (figure 4*c*). Here, a positive change in social status may be evaluated similarly to other motivators of animal and human behaviour such as food and money [19–21]. Previous work has shown reinforcement learning signals in the striatum during social tasks that are analogous to signals observed in non-social human decision-making tasks [24,34–37]. In particular, Izuma *et al*. [36] have shown that social rewards and monetary rewards are similarly processed in the human striatum. And it has been shown that a response in the dorsal striatum during a social exchange task shifts temporally in a manner similar to that seen in reward prediction errors for simple rewards [19–21,37]. Our present results add to a developing framework in which social signals are handled in the human brain much like more basic rewards that can be explained by mathematically specified mechanisms of social reward signals [34]. Importantly, this result also suggests that the LP group does not show a diminished valuation of social rank, which is consistent with motivation in this task being similar to their HP counterparts.

In the preceding analyses and discussion, we assess neurobehavioural correlates that distinguish HP and LP individuals, which showed a significant skewing in the distribution of male and female subjects (10/13 male in HP and 11/14 female in LP, two-tailed *p*-value = 0.007, using Fisher's exact test). One concern is that observed neurobiological differences (brain imaging responses) may be confounded by the gender imbalance between the two groups. This potential is an open point of discussion, which can only be settled empirically with future experiments. For instance, the same task could be performed with small groups consisting of a uniform gender. One would expect that if the responses observed were not gender specific then LP males in an all male group would express the same neurobehavioural characteristics as the LP group shown here, which was predominately composed of female subjects. Likewise, HP females in an all female group would express the same characteristics as the HP group presented here. This hypothetical result would be expected because the results presented here are derived from HP and LP groups that are each composed of male *and* female brains. Additionally, the general contrast of HP versus LP at ‘question reveal’ or ‘rank reveal’ did not show any significant clusters even at significance thresholds that are lower than accepted standards. This suggests that the gender imbalance is not sufficient to identify gross differences in brain responses during the task. It is only when we reduce the behavioural space by specifically looking for signals that change over time in all subjects and follow up that analysis with a more detailed look (i.e. ROI time-series analysis), that the differences in responses between the two groups are revealed (i.e. differences in amygdala and LPFC). Finally, we observe responses in the nucleus accumbens that are not different across HP and LP groups, consistent with similar responses across male and female brains in this task in at least one important region. The interpretation of these results does not rule out that gender may be a major factor in the observed behaviour and brain responses as the groups diverge in this task. However, prior to the work presented here there was no clear *a priori* reason to believe that male and female brains matched for paper and pencil IQ scores would demonstrate such a difference in our task. Our analyses have revealed an important observation that requires development in future work investigating the neurobehavioural effects of implicit signalling in small groups.

We have demonstrated a significant effect of socially relevant signals on individual behaviour and expression of cognitive capacity, which was estimated by performance on the ranked group IQ task. The extent to which these effects are present in real-world settings is unknown; however, it is clear that society at the levels of small and large culturally defined groups, act on analogous performance-based filters. Given the harmful effect demonstrated here and the correlation with specific neural signals, future research should determine at least three questions: (i) what exactly is society selecting for in competitive learning and workplace environments, which implicitly rank individuals on a myriad of dimensions, (ii) what are the causal relationships between the behaviour and neural responses observed in the present work, and (iii) can individuals overcome the observed negative influence of signals about social status and group membership? Regarding the last issue, the possibility that signals about group membership and low status can diminish individuals' abilities to express intelligent decision-making and problem solving ability suggests a real biological hypothesis about how conflict between large groups may manifest in violence, which would not otherwise be acceptable in interpersonal disagreements. Further work is needed to understand the neurobehavioural effects of diffusing group membership such that the individual is regained and the effect virtual group dissolution has on resolving intergroup conflict.
